# Comparative Performance of Four Electrodes for Measuring the Electromechanical Response of Self-Damage Detecting Concrete under Tensile Load

**DOI:** 10.3390/s19173645

**Published:** 2019-08-21

**Authors:** Hyeon Woo Noh, Min Kyoung Kim, Dong Joo Kim

**Affiliations:** Department of Civil and Environmental Engineering, Sejong University, 209, Neungdong-ro, Gwangjin-gu, Seoul 05006, Korea

**Keywords:** self-sensing, attached electrode, self-damage detecting concrete, copper material, carbon material

## Abstract

Self-damage or/and stress-sensing concrete is a promising area of research for measuring the electromechanical response of structural materials using more robust sensors. However, the copper and silver paste sensors widely used in such applications can be expensive and have detrimental effects on the load carrying capacity and durability of the structural systems upon which they are installed. Accordingly, this study compared the performance of four electrode types—conventional copper tape with silver paste (CS), copper film with type 1 carbon tape (CC1), copper film with type 2 carbon tape (CC2), and copper wire and film with type 2 carbon tape (WC2)—to develop an economical and practical electrode for measuring the electromechanical response of self-damage-detecting concrete. The CC1 electrode exhibited comparable performance to the CS electrode in measuring the electromechanical response of self-damage-detecting concrete, despite requiring a longer polarization time (80 s) than the CS electrode (25 s). The CS electrode exhibited a higher damage-sensing capacity (GF_2_), whereas the CC1 electrode exhibited a higher strain-sensing capacity (GF_1_), as well as good damage-sensing capacity. Therefore, the CC1 electrode using copper film with type 1 carbon tape was determined to be the best alternative to the conventional CS electrode.

## 1. Introduction

Catastrophic collapses of civil infrastructure and buildings have brought about increased attention to the necessity of structural health monitoring (SHM) systems [1,2,3]. Current SHM systems mostly utilize embedded and/or attached sensors [4,5,6], but these types of sensors have exhibited relatively low durability and very limited sensing capacity. Accordingly, over the last two decades, much research has been performed on the self-sensing capacity of cement-based construction materials containing conductive fillers, such as steel fibers, carbon fibers, multi-walled carbon nanotubes, carbon black, and graphite powder under various loads [7,8,9,10,11,12,13,14,15,16,17,18,19,20,21,22,23]. The electrical impedance or resistivity of self-sensing concrete containing electrically conductive fillers is well-known to change under stress and/or due to the occurrence of damage, including cracks. In this application, it is important to correctly measure the pure electrical response of the self-sensing concrete under load.

The correct evaluation of damage or stress based on the electromechanical response of self-damage and/or stress-sensing concrete requires suitable electrodes that have the following properties recommended by Han et al. [24] and Azhari [25]: electrode materials should be electrically conductive and durable under severe environmental exposure and repeated mechanical loads; the addition of the electrode should not negatively affect the concrete strength; and the electrode should be easily connected to a data acquisition system. Azhari [25] recommended copper and silver paste as suitable electrode materials. Indeed, many researchers have used copper and silver paste in electrodes when investigating the electro-mechanical responses of various self-sensing concretes [13,15,17,26,27,28,29,30]. However, current embedded-type electrodes utilizing copper materials can possibly generate negative effects on both the load-carrying capacity and durability of the structural systems that contain them. Additionally, attached-type electrodes utilizing silver paste are very expensive and are not reusable. A suitable electrode should be highly conductive, affordable, and easily attached to the surface of self-damage and/or stress-sensing concrete.

The objective of this study is accordingly to determine a suitable electrode for the measurement of the electromechanical response of self-damage detecting concrete by utilizing or combining common electrically conductive materials. The specific objectives are (1) to investigate the electrical polarization depending on the type of electrode, and (2) to measure the electromechanical response of self-damage-detecting concrete under tensile load using the evaluated electrodes.

## 2. Current Electrodes

### 2.1. Embedded-Type Electrodes

Current embedded-type electrodes mostly include steel or copper wire mesh or wire gauze consisting of grid structures. Han and Ou [30] embedded copper wire meshes in self-sensing concrete containing carbon-based fillers to measure the electrical response under repeated compressive loads. They reported an 8% decrease in the electrical resistivity of self-sensing concrete containing these carbon-based fillers. Sun et al. [31] utilized steel wire meshes as embedded electrodes to measure the electromechanical responses of self-sensing concretes containing graphite. As the magnitude of the applied compressive load increased, the electrical resistivity of the graphite-reinforced self-sensing concretes decreased—the reduction in electrical resistivity was 15.6%. Both steel and copper wire meshes demonstrated good performance as embedded type electrodes. Azhari [25] investigated the effects of curing time on the electrical resistivity of cement-based materials by using different electrodes (copper plate, copper mesh, and copper wire with silver paste)—the embedded-type electrodes (copper plate and copper mesh) produced a smaller change in the measured electrical resistivity than the attached-type electrode (copper wire with silver paste).

### 2.2. Attached-Type Electrodes

Current attached-type electrodes generally utilize silver paste, silver paint, copper wire, or copper tape. Song et al. [15] applied an attached-type electrode using copper tape and silver paste on the surface of high performance fiber-reinforced cementitious composites (HPFRCCs) to measure their electromechanical response during direct tensile tests. They reported that the electrical resistivity of the HPFRCCs clearly decreased as the number of micro-cracks increased within the gauge length. Azhari and Banthia [13] also used attached-type electrodes with copper wire and silver paste to measure the electromechanical response of self-sensing concretes containing carbon materials, which exhibited a noticeable decrease (25%) in the electrical resistivity under repeated compressive load.

Han et al. [24] compared the electrical polarization of an embedded-type electrode (copper gauze) and an attached-type electrode (copper foil). The embedded electrode generated a lower polarization potential than the attached electrode, although both electrodes produced different polarization potentials corresponding to different amounts of current. They reported that the polarization potential was affected by the different effective areas between the electrode and the matrix—copper foil with a larger effective area exhibited higher polarization potential than copper gauze with a lower effective area. Thus, different electrodes have been observed to produce different electrical and/or electromechanical responses in cement-based composites according to their different geometric and material properties.

## 3. Experimental

**Figure 1:**
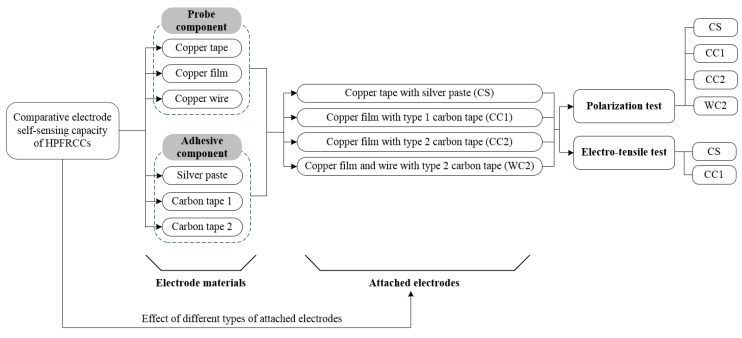
Experimental program. HPFRCCs = high performance fiber-reinforced cementitious composites.

The experimental program in this study, described in Figure 1, was designed to compare the performance of four different electrodes when measuring the electrical response of self-damage detecting concrete. The configuration of the electrode can be divided into two components: the probe component, which measures the electrical impedance or resistance of the self-damage detecting concrete, and the adhesive component, which transfers the applied electrical current into the self-damage-detecting concrete. Materials evaluated in this study included copper film and copper wire, and two different types of carbon tape were evaluated as the adhesive component. 

Table 1 summarizes the properties of the electrode materials evaluated in this study. By combining different materials in the probe and adhesive components, three types of electrode were investigated as follows: 1) copper film with type 1 carbon tape (CC1); 2) copper film with type 2 carbon tape (CC2); and 3) copper wire and film with type 2 carbon tape (WC2). A conventional electrode consisting of copper tape with silver paste (CS) was also evaluated as a control. The performance of each electrode was evaluated by measuring the polarization effect and by investigating the electromechanical response of the HPFRCCs under tension.

### 3.1. Materials and Specimen Preparation

Specimens of HPFRCC containing 1 vol% long and 1 vol% medium smooth steel fibers were investigated to evaluate the performance of the three electrodes (CC1, CC2, and WC2). The composition of the matrix and its compressive strength are provided in Table 2. The diameter of the silica sand was 0.36 mm on average and the water-to-cement ratio was 0.35. The compressive strength of the matrix was measured to be 95 MPa by 100 × 200 mm cylinder specimen tests. The properties of the steel fibers are summarized in Table 3—the length and diameter of the long smooth steel fibers were 30 and 0.3 mm, respectively, while those of the medium smooth steel fibers were 19.5 and 0.2 mm, respectively.

A 20 L capacity Hobart-type laboratory mixer was used to mix the specimens. Cement, silica sand, and fly ash were first dry mixed for 10 min. Then, water was added and further mixed for 5 min. When the mortar mixture exhibited suitable workability and viscosity to provide uniform fiber distribution, the shorter fibers were first carefully dispersed, then the longer fibers were added in by hand. The mortar mixture with the fibers was then further mixed for 1–2 min.

**Figure 2:**
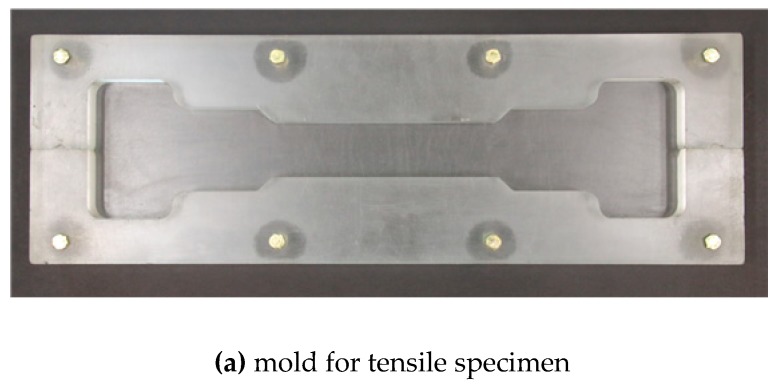

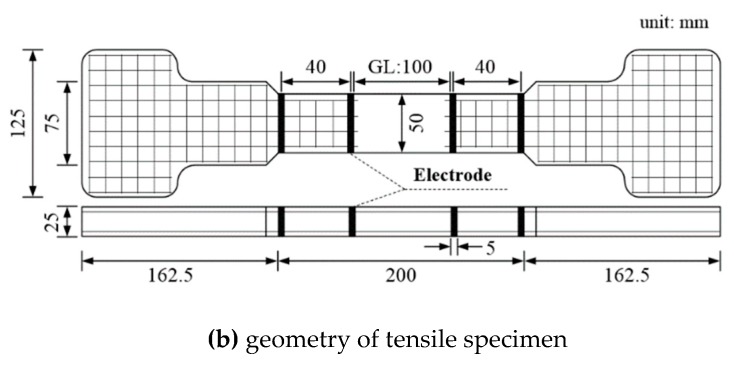
Tensile specimen mold and geometry.

The mortar mixture containing the steel fibers was poured into molds for both the polarization and tensile test specimens, as can be seen in Figure 2a. Two layers of steel wire mesh were used to reinforce both ends of the specimen, as can be seen in Figure 2b. The specimens were then covered with a plastic sheet and kept in a laboratory at room temperature for 24 h. Next, the specimens were demolded and cured in water for 2 weeks. After curing, the specimens were kept in dry conditions for 2 h, then the surfaces of the specimens were ground to accommodate the attachment of electrodes. At least three specimens (SP1, SP2, and SP3) were prepared for each electrode test series.

### 3.2. Test Set-Up and Procedure

**Figure 3:**
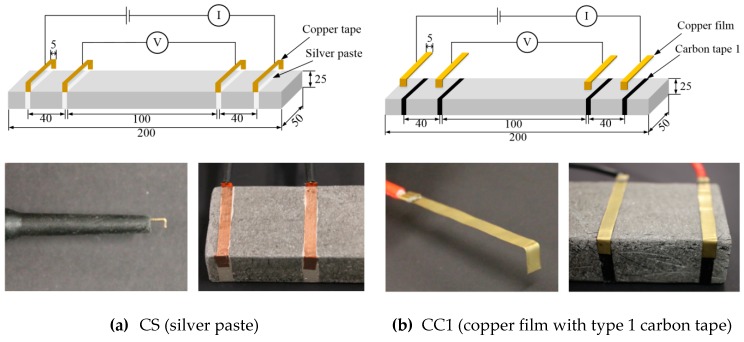

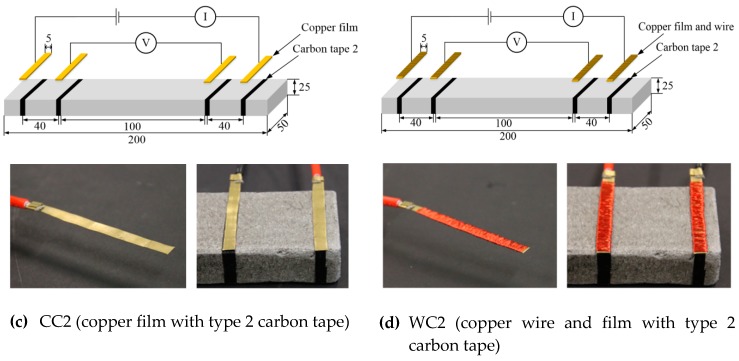
Electrode arrangement with different types of electrodes.

The four-probe measurement method proposed by Wenner [32], which requires two outer input current electrodes and two inner output current electrodes, was employed to measure the electrical polarization and electromechanical response of the self-damage-detecting concrete, as shown in Figure 3. The distance between the two inner electrodes was 100 mm, while that between the inner and outer electrodes was 40 mm. The geometry of tensile specimens and test set-up in this study were referred to relevant previous studies [17,33].

**Figure 4:**
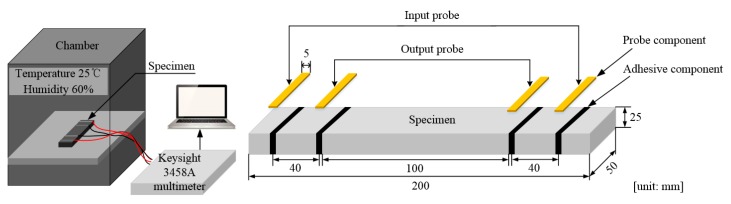
Polarization effect stabilization test set-up and details about the specimen.

A commercial multimeter (Keysight 3458A, Santa Rosa, California, USA) was used to measure the change in electrical resistance during the tests. The electrical resistivity of the HPFRCCs was measured by using direct current (DC) measurements to obtain a more accurate electrical resistivity [34]. The magnitude of the input current was maintained at 50 μA for 14 min to monitor the electrical polarization. During the polarization tests, the specimens were kept in a chamber with a constant temperature (25 °C) and humidity (60%). The test setup is illustrated schematically in Figure 4.

**Figure 5:**
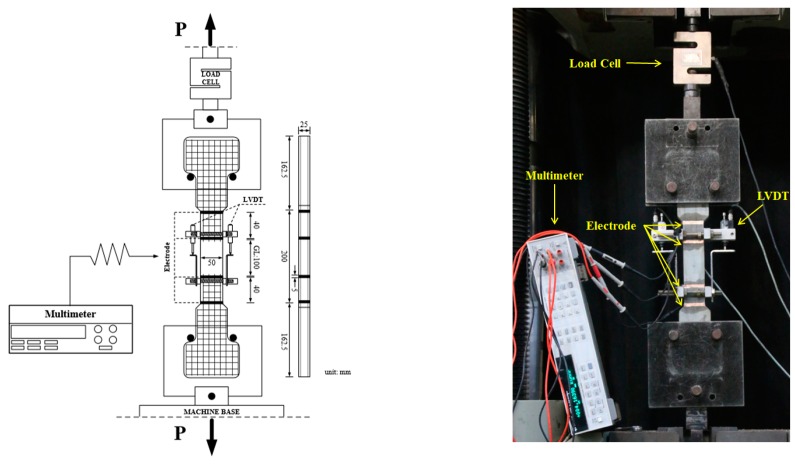
Set-up for measuring the electrical resistance of HPFRCCs during direct tensile tests. LVDT = linear variable differential transformers.

Figure 5 shows the test set-up for measuring the electrical resistance of the HPFRCCs during the direct tensile tests. A universal testing machine (UTM) was used to apply a constant 1.0 mm/min rate of displacement. The tensile elongation of the specimens was measured by two linear variable differential transformers (LVDTs) installed in an aluminum cage, while the tensile stress was measured by a 50 kN capacity load cell. To measure the electrical resistance of the specimens under tension, the Keysight 3458A multimeter was connected to the specimen, as shown in Figure 5. Prior to tensioning, the electrical resistance was measured for at least 15 min to stabilize the effects of electrical polarization.

## 4. Results and Discussion

**Figure 6:**
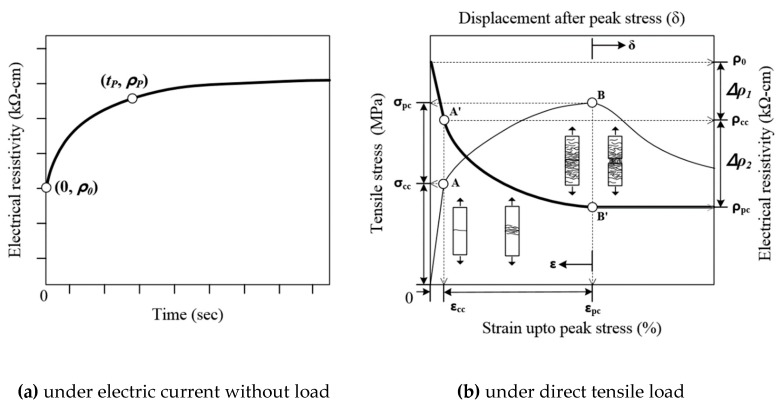
Typical change of electrical resistivity of HPFRCCs.

Figure 6a,b shows the typical change in electrical resistivity of the HPFRCCs due to electrical polarization before applying tensile load and under direct tension, respectively. In Figure 6a, under electric current without load, the electrical resistivity can be observed to rapidly increase until reaching the polarization time required for stable electrical resistivity. In Figure 6b, it can be observed that as the tensile strain (ε) increased from 0 to the first cracking strain (ε_cc_), the tensile stress (σ) linearly increased from 0 to the first cracking strength (σ_cc_, point A) of the HPFRCCs under direct tension, while the electrical resistivity (ρ) decreased from the initial electrical resistivity (ρ_0_) to that at the first cracking point A’ (ρ_cc_). Even though the slight change in electrical resistance within the elastic limit prior to points A and A’ cannot be clearly correlated to the change in applied tensile stress, the electrical resistivities of the HPFRCCs beyond points A and A’ significantly decreased until the post-cracking points B and B’, as illustrated in Figure 6b. The electrical resistivity considerably decreased from ρ_cc_ (point A’) to the post-cracking electrical resistivity at point B’ (ρ_pc_), while the tensile strain increased from ε_cc_ to the post-cracking strain (ε_pc_), and the tensile stress increased from σ_cc_ to the post-cracking strength (σ_pc_, point B). Kim et al. [17] reported that the electrical resistance of HPFRCCs under direct tension with multiple micro-cracks is composed of a non-cracked component and a cracked component. Since the electrical conductivity of the steel fibers in the cracked portion of an HPFRCC is much higher than that in the non-cracked portion, the electrical resistance decreases as the number of micro-cracks increases.

The electrical resistivity (ρ) was calculated using the following Equation (1):

(1) $$\rho =R\frac{A}{L},$$

where ρ is electrical resistivity (kΩ·cm), R is the electrical resistance (kΩ), A is the cross-sectional area of the specimen (cm^2^), and L is the distance between the input and output electrodes (cm).

**Figure 7:**
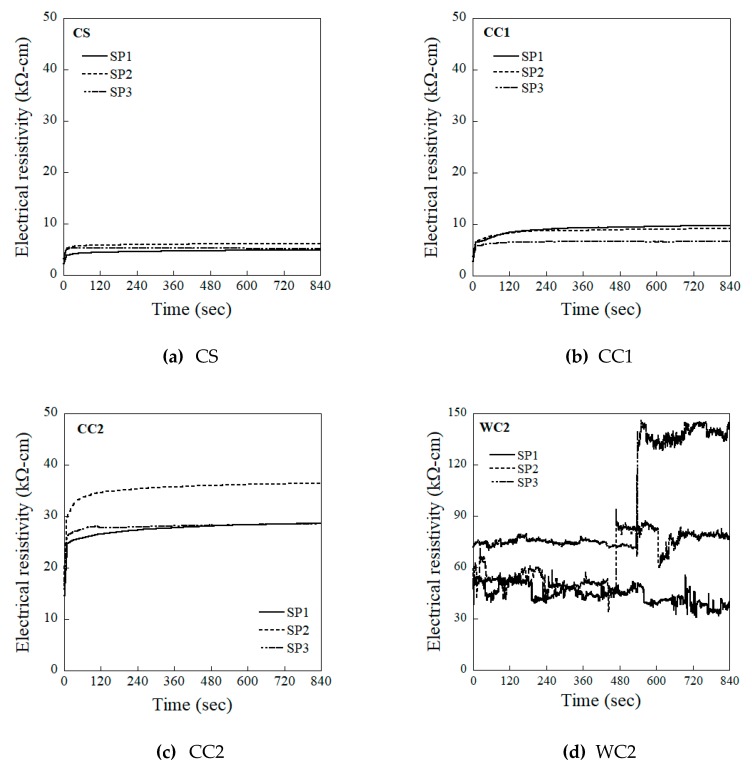
Electrical resistivity history with time for HPFRCCs with different types of electrode (calibrated 0.1 Hz).

Figure 7 shows the electrical resistivity history of identical HPFRCCs for 840 s (14 min) prior to loading, measured using the four evaluated electrode types (CS, CC1, CC2, and WC2) under electric current without any applied load. The multimeter was calibrated to collect data only within a range of 6 to 0.1 Hz to minimize noise. The electrical responses measured using the CS, CC1, and CC2 electrodes clearly show the typical electrical polarization phenomena for 840 s, as can be seen in Figure 7, while the response measured using the WC2 electrode did not. Among the three electrodes that exhibited a typical polarization response, the CS electrode exhibited the shortest polarization time (25 s). Based on the polarization tests, CC2 and WC2 electrodes were excluded in the investigation of the electromechanical response of HPFRCCs under direct tensile load, as both electrodes produced significant polarization and electrical noise.

**Figure 8:**
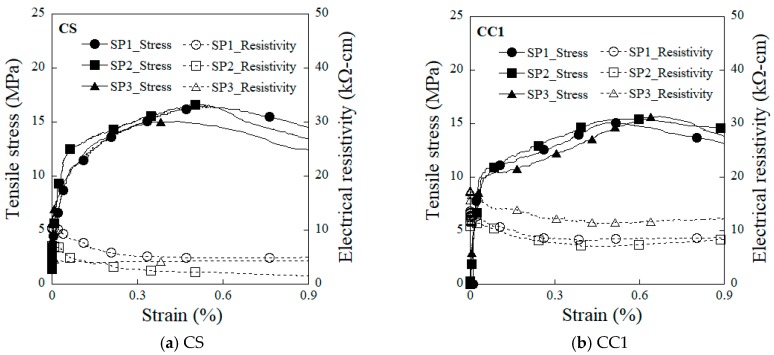
Electro-tensile behavior of HPFRCCs with different types of electrodes.

Figure 8 shows the electromechanical responses of the HPFRCCs under direct tensile load as measured using electrodes types CS and CC1, both of which exhibit the typical electromechanical response of HPFRCCs with steel fibers. To quantify the strain- and damage-sensing capacity of the HPFRCCs, their gauge factors (GF, GF_1_, and GF_2_) were calculated using the following Equation (2) [17]:

(2) $$GF=\frac{\Delta \rho }{\rho_{0}\cdot \varepsilon_{pc}};\;\;\;GF_{1}=\frac{\Delta \rho_{1}}{\rho_{0}\cdot \varepsilon_{cc}};\;\;GF_{2}=\frac{\Delta \rho_{2}}{\rho_{cc}\cdot (\varepsilon_{cc}-\varepsilon_{pc})},$$

where GF_1_ represents the strain-sensing capacity within the elastic range prior to first cracking, GF_2_ denotes the damage-sensing capacity of the HPFRCC from after first cracking to the post-cracking point, and the overall sensing-capacity can be estimated using GF. Specimen SP1 of the HPFRCC using the CS electrode was excluded from the calculation of the average GF values due to the large deviation of its test results.

The HPFRCCs using the CC1 electrode exhibited a higher strain-sensing capacity (GF_1_ = 2.39) than those using the CS electrode, as well as good damage-sensing capacity (GF_2_ = 0.65). Consequently, the electromechanical response of the HPFRCCs measured using the CC1 electrode generated comparable self-strain and damage-sensing capacity to that measured using the conventional CS electrode.

### 4.1. Effects of Different Electrodes on the Polarization Parameters

**Figure 9:**
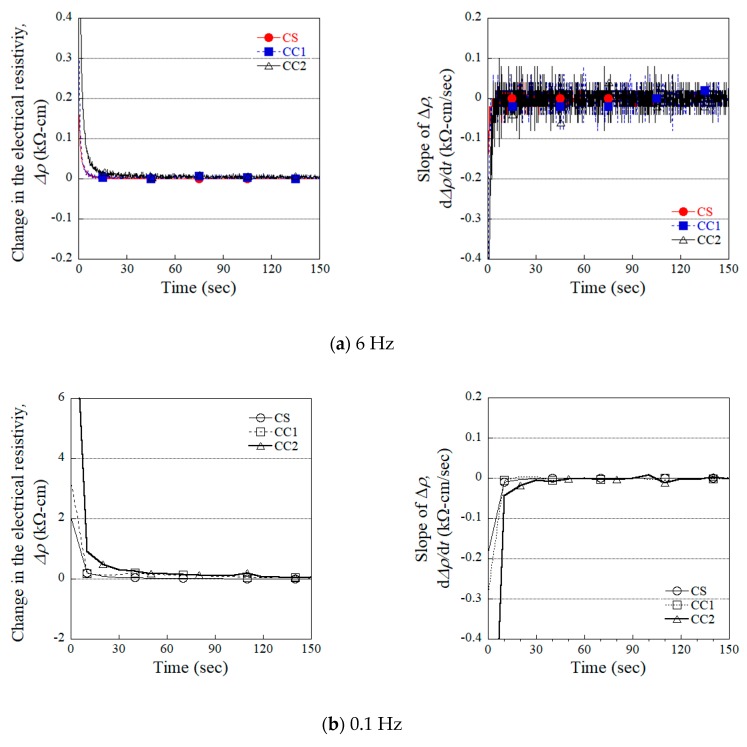
Change in the electrical resistivity and slope of change in the electrical resistivity with calibrated different data frequency.

Table 4 summarizes the parameters describing the electrical polarization corresponding to the four different evaluated electrodes. These polarization parameters consist of the initial electrical resistivity (ρ_0_), electrical resistivity at polarization time (ρ_P_), fractional change in the electrical resistivity from ρ_0_ to ρ_P_ ($\bar{\rho_{p}}$), change in the electrical resistivity at polarization time (∆ρ_P_), slope at polarization time (ρ’_P_), and polarization time (t_p_). Figure 9 shows the measured change in the electrical resistivity (∆ρ) and the variation in the slope (ρ’) of that change corresponding to the different types of electrodes evaluated, used to determine the polarization time (t_p_) required to establish stable electrical resistivity, defined in this study as the time satisfying both of the following conditions: 1) when ∆ρ is less than 0.09 kΩ·cm, and 2) when ρ’ is less than 0.009 kΩ·cm/sec. To minimize noise when determining t_p_, the data measured at a frequency of 6 Hz was calibrated to the data measured at a frequency of 0.1 Hz, as can be seen in Figure 9a,b. The above conditions for determining t_p_ could then be obtained using the measured data calibrated to 0.1 Hz. As a result, the t_p_ for the specimens equipped with the CS, CC1, and CC2 electrodes was determined to be 25 s, 80 s, and 107 s, respectively.

**Figure 10:**
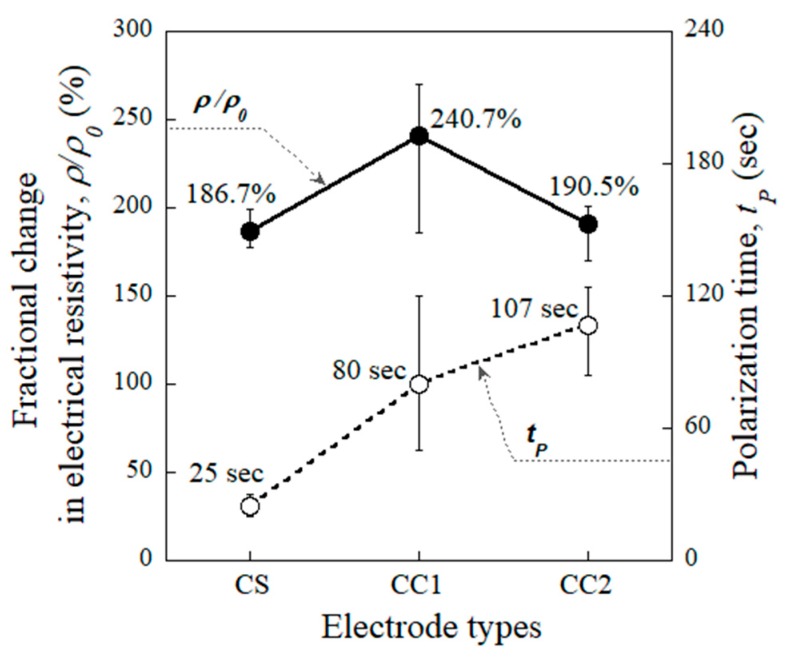
Relationship between the fractional change in the electrical resistivity and polarization time of HPFRCCs corresponding to the electrode types.

Figure 10 shows the relationships between the fractional change in the electrical resistivity and polarization time of the CS-, CC1-, and CC2-equipped HPFRCCs. The fractional changes in the electrical resistivity at the polarization time ($\bar{\rho_{p}}$) of the CS-, CC1-, and CC2-equipped HPFRCCs were 186.7%, 240.7%, and 190.5%, respectively. The value of $\bar{\rho_{p}}$ can be observed to be closely related to the value of t_p_—the $\bar{\rho_{p}}$ of the CS- and CC1-equipped HPFRCCs increased from 186.7% to 240.7%, as the t_p_ increased from 25 s to 80 s.

**Figure 11:**
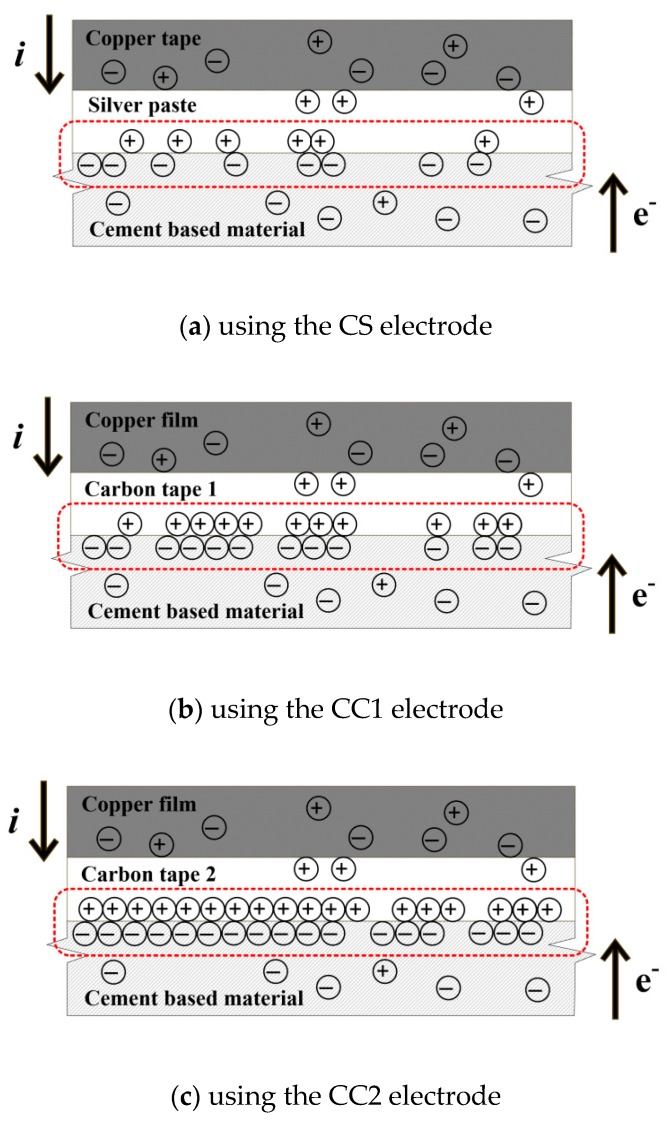
Accumulation phenomenon of electrons at the interface between the probe and HPFRCCs.

The correlation between $\bar{\rho_{p}}$ and t_p_, describing the polarization phenomenon, can be explained by the electron flow at the interface between the electrode probes and the HPFRCCs. Suryanto et al. [35] reported that the electrical resistance of cementitious composites was affected not only by the matrix characteristics (compressive strength, temperature, humidity, etc.) but also the electrical resistance of the interface. Figure 11a–c illustrates the electron accumulation phenomenon at the interface between each electrode type and the HPFRCCs. When the input current (i) flows from the probe into the cement based material, negative electrons (e^-^) move in the opposite direction [36]. At this time, electron accumulation, which causes the polarization effect, occurs between the probe (which has a high conductivity) and the cement based material (which has a low conductivity). As the electron accumulation increases, both the polarization time (t_p_) and the fractional change in the electrical resistivity at polarization time ($\bar{\rho_{p}}$) increase. Therefore, as can be seen in Figure 11a, electrons at the interface between the CS electrode and the HPFRCC, which exhibited a shorter t_p_, accumulated less than for other electrodes, whereas the electrons at the interface between the CC2 electrode and the HPFRCC, which exhibited a longer t_p_, accumulated more, as can be seen in Figure 11c. Consequently, the $\bar{\rho_{p}}$ and t_p_ of the CC1-equipped HPFRCC, which exhibits more electron accumulation between the specimen and the electrode than the CS-equipped HPFRCC, were higher than those of the CS-equipped HPFRCC. The results obtained using the CC2 electrode showed the highest t_p_ (107 s), and a $\bar{\rho_{p}}$ (190.5%) lower than that when using CC1 electrode, as the CC2 electrode (which used a smaller-area carbon tape as the adhesive) seemed to generate significantly more electron accumulation at the interface between the specimen and the electrode. Thus, the CC2 electrode was determined to be unsuitable for measuring the electrical resistance of HPFRCCs. Among the evaluated electrodes, the CS electrode exhibited the shortest t_p_ (25 s). Among the remaining electrodes, because electrode CC1 exhibited a shorter t_p_ (80 s) than electrode CC2 (107 s), CC1 was determined to be more suitable than CC2 for measuring electrical polarization. Accordingly, the CS and CC1 electrodes were selected as the focus of the remaining investigation.

### 4.2. Effects of Electrode Types on Electromechanical Response

Table 5 summarizes the electromechanical response parameters (ε_cc_, σ_cc_, ε_pc_, σ_pc_, ρ_0_, ρ_cc_, and ρ_pc_) of HPFRCCs corresponding to the two most effective electrode types evaluated in this study (CS and CC1). All specimens, regardless of the type of electrode used, exhibited a tensile strain-hardening response and similar mechanical resistance. The average first-cracking strength (σ_cc_) was 5.5 MPa and 6.4 MPa for the CS- and CC1-equipped HPFRCCs, respectively; the average post-cracking strength (σ_pc_) was 14.9 MPa and 15.7 MPa, respectively; and the average strain capacity (ε_pc_) was 0.48% and 0.56%, respectively.

**Figure 12:**
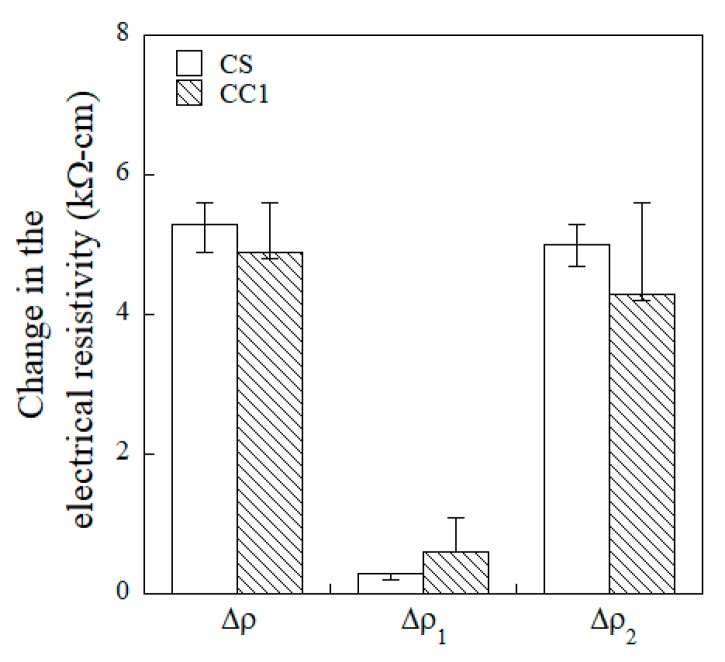
Change in the electrical resistivity of HPFRCCs with CS and CC1 electrodes under direct tensile load.

There was clear difference in the measured initial electrical resistivity (ρ_0_) of the CS- and CC1-equipped HPFRCCs, which were 8.8 kΩ·cm and 14.1 kΩ·cm, respectively. However, as can be seen in Figure 12, the overall change in electrical resistivity (∆ρ) was 5.3 kΩ·cm and 4.9 kΩ·cm for the CS- and CC1-equipped HPFRCCs, respectively, even though the materials used in the CS electrode exhibited a higher electrical conductivity than those used in the CC1 electrode.

Table 6 compares the effects of electrode type on the gauge factors. The CS-equipped HPFRCCs showed a higher damage-sensing capacity (GF_2_), whereas the CC1-equipped HPFRCCs exhibited a higher strain-sensing capacity (GF_1_), as well as good damage-sensing capacity—GF_1_ of the CC1-equipped HPFRCC was 2.39 and its GF_2_ was 0.65. Therefore, the CC1 electrode using copper film with type 1 carbon tape was identified as the best suited for use as a replacement for the conventional copper tape with silver paste (CS) electrode in measuring the electromechanical response of HPFRCCs.

## 5. Conclusions

In this study, we investigated the electromechanical response performance of four electrodes for use in self-damage detecting concrete: a conventional copper tape with silver paste (CS), copper film with type 1 carbon tape (CC1), copper film with type 2 carbon tape (CC2), and copper wire and copper film with type 2 carbon tape (WC2). The CC1 electrode exhibited the best performance among the three types of electrodes investigated to replace the conventional CS electrode. On the basis of the experimental results, the following conclusions can be drawn:

- Both the CC1 and CS electrodes produced a relatively shorter polarization time than the other electrodes (CC2 and WC2). The polarization times (t_p_) for the specimens using the CS and CC1 electrodes were 25 s and 80 s, respectively.
- The t_p_ was significantly affected by the accumulation of electrons at the interface between the specimen and electrode. Higher levels of electron accumulation resulted in longer polarization times.
- The overall change in the electrical resistivity (∆ρ) upon damage to specimens equipped with the CS and CC1 electrodes was 5.3 kΩ·cm and 4.9 kΩ·cm, respectively. The CS-equipped HPFRCCs showed a higher damage-sensing capacity (GF_2_), whereas the CC1-equipped HPFRCCs exhibited a higher strain-sensing capacity (GF_1_), as well as good damage-sensing capacity.
- The CC1 electrode using copper film with type 1 carbon tape is identified as the best suited for use as a replacement for the conventional copper tape with silver paste (CS) electrode in measuring the electromechanical response of self-damage-detecting concrete.

We determined that the CC1 electrode can be used as a replacement for the conventional copper tape with silver paste (CS) electrode under direct tensile load. This is an improvement in economic terms as well, as using carbon tape is a third of the cost of using silver paste. In future research, we intend to demonstrate the suitability of the CC1 electrode for use with HPFRCCs under compressive load, cyclic load, and other measuring conditions (using alternating current, biphasic direct current, multiple electrodes, the two probe method, reusability and accounting for temperature influence, etc.) to more completely capture the capabilities and performance of this electrode type.

## Figures and Tables

**Table 1:** Properties of electrode materials.

Electrode Material	Thickness, t (mm)	Width, w (mm)	Length, l (mm)	Cross-Section, A (mm^2^) ^†^	Electrical Resistivity (× 10^−7^ kΩ·cm)
Copper tape	0.12	10	100	1.20	0.058 ^a^
Copper film	0.30	5	50	1.50	0.023 ^b^
Carbon tape type 1	0.16	10	100	1.60	1.3 ^a^
Carbon tape type 2	0.18	5	100	0.90	1.3 ^a^
Copper wire	0.16 *		1000	0.02	0.000017 ^a^

^†^ A = t × w, * diameter of copper wire, ^a^ manufacturer obtained the measured electrical resistivity, ^b^ measured electrical resistivity using the four probe method.

**Table 2:** Matrix composition and compressive strength.

Cement (Type III)	Silica Sand (#40) *	Fly Ash	Super-Plasticizer **	Water	Compressive Strength (MPa)
1.0	1.0	0.15	0.0055	0.35	95

* average particle size of silica sand is 0.355 mm, ** super-plasticizer contained 25% solid content.

**Table 3:** Properties of steel fibers.

Fiber Type	Diameter (mm)	Length (mm)	Density (g/cc)	Tensile Strength (MPa)	Elastic Modulus (GPa)	Electrical Resistivity (× 10^−10^ kΩ·cm) *
Long smooth	0.3	30	7.90	2447	200	6.9
Medium smooth	0.2	19.5	7.90	2942	200	5.4

* measured electrical resistivity using four probe method.

**Table 4:** Polarization response parameters of HPFRCCs with different types of electrodes. SP = specimen.

Type of Electrode	No.	Electrical Resistivity (kΩ·cm)			Fractional Change in Electrical Resistivity (%)	Slope at Polarization Time (kΩ·cm/s) *	Polarization Time (s)
		ρ_0_	ρ_P_	∆ρ_P_	$\bar{\rho_{p}}$	d∆ρ_P_/dt_p_	t_p_
CS	SP1	2.2	4.0	0.10	190.8	0.0020	30
	SP2	3.2	5.5	0.12	173.8	0.0020	30
	SP3	2.7	5.3	0.06	195.5	0.0040	20
	Avg. ^a^	2.7	4.9	0.09	186.7	0.0027	25
	STD ^b^	0.4	0.7	0.02	9.3	0.0009	4.7
CC1	SP1	2.8	8.1	0.13	295.8	0.0010	110
	SP2	3.8	8.1	0.08	211.3	0.0000	90
	SP3	2.8	6.4	0.04	214.9	0.0000	40
	Avg.	3.2	7.5	0.08	240.7	0.0000	80
	STD	0.5	0.8	0.04	39.0	0.0005	29.4
CC2	SP1	14.6	26.5	0.07	180.3	0.0010	100
	SP2	16.5	34.6	0.11	210.6	0.0010	130
	SP3	15.5	27.7	0.04	180.5	0.0060	90
	Avg.	15.5	29.6	0.07	190.5	0.0027	107
	STD	0.8	3.6	0.03	14.2	0.0024	17.0
WC2	SP1	51.0	-				
	SP2	48.8					
	SP3	71.3					
	Avg.	57.0					
	STD	10.1					

* absolute value of the calculated value, ^a^ Avg.: average values; ^b^ STD: standard deviation.

**Table 5:** Electro-tensile parameters of HPFRCCs with CS and CC1 electrodes.

Notation		Tensile Strain (%)		Tensile Stress (MPa)		Electrical Resistivity (kΩ-cm)			Change in the Electrical Resistivity (kΩ-cm)		
		ε_cc_	ε_pc_	σ_cc_	σ_pc_	ρ_0_	ρ_cc_	ρ_pc_	∆ρ	∆ρ_1_	∆ρ_2_
**CS**	SP1	0.01	0.44	6.0	13.3	5.1	4.3	4.2	0.9	0.8	0.1
	SP2	0.02	0.48	6.0	15.8	7.1	6.9	2.2	4.9	0.2	4.7
	SP3	0.02	0.51	4.5	15.4	10.4	10.2	4.9	5.6	0.3	5.3
	Avg.	0.02	0.48	5.5	14.9	8.8	8.5	3.5	5.3	0.3	5.0
	STD	0.00	0.02	0.75	0.20	1.65	1.65	1.35	0.35	0.05	0.30
CC1	SP1	0.03	0.50	5.3	15.1	12.8	12.7	8.5	5.2	1.0	4.2
	SP2	0.02	0.54	6.4	15.5	12.1	11.4	7.3	4.8	0.6	4.2
	SP3	0.03	0.65	7.4	15.7	17.4	16.4	11.9	5.6	1.1	5.6
	Avg.	0.03	0.56	6.4	15.4	14.1	13.5	9.2	4.9	0.6	4.3
	STD	0.00	0.06	0.86	0.25	2.35	2.12	1.95	0.33	0.22	0.66

**Table 6:** Gauge factors of HPFRCCs with CS and CC1 electrodes under direct tensile load.

Notation		Overall Sensing Capacity, GF	Strain-Sensing Capacity, GF_1_	Damage-Sensing Capacity, GF_2_
CS	SP1 ^¥^	0.40	15.69	0.05
	SP2	1.44	1.41	1.48
	SP3	1.06	1.44	1.06
	Avg.	1.22	1.43	1.23
	STD	0.19	0.02	0.21
CC1	SP1	0.81	2.60	0.70
	SP2	0.73	2.48	0.71
	SP3	0.50	2.11	0.56
	Avg.	0.62	2.39	0.65
	STD	0.13	0.21	0.07

^¥^ the specimen 1 (SP1) of the HPFRCCs using the CS electrode was excluded in the calculation of the average.
